# Graphene-Encapsulated Silver Nanoparticles for Plasmonic Vapor Sensing

**DOI:** 10.3390/nano12142473

**Published:** 2022-07-19

**Authors:** Gábor Piszter, György Molnár, András Pálinkás, Zoltán Osváth

**Affiliations:** Centre for Energy Research, Institute of Technical Physics and Materials Science, 1121 Budapest, Hungary; gabor.piszter@ek-cer.hu (G.P.); gyorgy.molnar@ek-cer.hu (G.M.); andras.palinkas@ek-cer.hu (A.P.)

**Keywords:** silver nanoparticles, graphene coating, LSPR-based sensing, VOC detection

## Abstract

Graphene-covered silver nanoparticles were prepared directly on highly oriented pyrolytic graphite substrates and characterized by atomic force microscopy. UV–Vis reflectance spectroscopy was used to measure the shift in the local surface plasmon resonance (LSPR) upon exposure to acetone, ethanol, 2-propanol, toluene, and water vapor. The optical responses were found to be substance-specific, as also demonstrated by principal component analysis. Point defects were introduced in the structure of the graphene overlayer by O_2_ plasma. The LSPR was affected by the plasma treatment, but it was completely recovered using subsequent annealing. It was found that the presence of defects increased the response for toluene and water while decreasing it for acetone.

## 1. Introduction

Noble metal nanoparticles (NPs) are widely used for chemical and biological sensing because of their local surface plasmon resonance (LSPR) [1,2] and surface-enhanced Raman scattering (SERS) [3,4] properties. The LSPR produces sharp spectral absorption, which can be used to detect changes in the molecular environment near the surfaces of NPs by spectral shift detection [5,6,7]. Due to their enhanced interaction with light, gold (Au) and silver (Ag) nanoparticles have been intensively studied as promising plasmonic sensing systems [8,9,10,11,12,13,14]. Their LSPR can be tuned by adjusting the size, shape, dispersion, and uniformity of the NPs, as well as the dielectric constant of the detection medium [15,16,17,18,19,20,21,22,23]. Ag nanostructures are often considered the best material for plasmonics, as there are no interband absorptions and only minimum loss at optical frequencies [24]. They have an inherently more intensive and sharper plasmonic spectrum than Au nanoparticles, enabling them to offer superior sensing functionality. Silver, however, has poor stability under ambient conditions and forms silver sulfide on its surface. This causes morphological changes in Ag NPs and a significant decrease in the optical properties [25]. Conserving the high LSPR intensity is critically important in potential sensor applications. A common method to protect Ag NPs from environmental effects is to form core–shell structures where the Ag surface is passivated with either organic or inorganic shells (for recent reviews, see Refs. [26,27]). Graphene, as an atomically thin material with very low permeability [28,29,30], seems to be an ideal passivating coating to prevent the surface oxidation of Ag films and NPs [25,31,32]. It was demonstrated that graphene can improve the sensitivity of silver-based LSPR sensors and delay the oxidation process of Ag NPs effectively [33,34,35,36]. Nevertheless, it was also shown that oxygen can penetrate through the defects and grain boundaries of large-area graphene grown by chemical vapor deposition (CVD) [37].

The synthesis of Ag NPs can be accomplished using a variety of strategies [38] depending on the desired shape of the nanoparticles. Chemical reduction in a bottom-up approach is the most used method. Many factors influence the size and shape of the produced Ag NPs, including temperature, silver precursor concentration, the strength of the chemical interaction between the capping agent and the different silver crystallographic planes [39], and so on. In this work, we fabricate Ag NPs and graphene–silver nanoparticle hybrids directly on highly oriented pyrolytic graphite (HOPG) substrates using a straightforward approach described earlier [40]. Scanning electron microscopy (SEM), atomic force microscopy (AFM), and UV–Vis reflectance spectroscopy are used, respectively, to investigate the morphology, optical, and vapor sensing properties of the hybrid nanomaterial. We show that graphene-covered Ag NP samples display a pronounced optical response upon exposure to organic vapors (acetone, ethanol, 2-propanol, toluene, or water), which can be observed in the shift in the LSPR. This optical response to the volatile organic compounds (VOC) is proportional to the vapor concentration and is substance-specific. Furthermore, we show that the optical response can be tuned by introducing point defects into the structure of the graphene overlayer.

## 2. Experimental

### 2.1. Preparation of Graphene-Covered Silver Nanoparticles

Silver films of 7 nm nominal thickness were evaporated onto HOPG substrates at a background pressure of 5 × 10^−7^ mbar and an evaporation rate of 0.1 nm s^−1^. The deposited Ag films were covered with CVD graphene grown on copper foil (Graphenea), immediately after the opening of the evaporation chamber. We applied polymer tape as mechanical support to transfer large-area graphene samples. The copper foil was removed using an etchant composed of copper (II) chloride aqueous solution (20%) and hydrochloric acid (37%) in a 4:1 volume ratio. After rinsing and drying, the polymer tape with the graphene was pressed onto the evaporated Ag films. The tape was removed by lifting it with tweezers. To form nanoparticles, both bare and graphene-covered Ag thin films were annealed at 400 °C under an inert gas (Ar) atmosphere for 1.5 h. The obtained samples (samples #1 and #2) were characterized by tapping-mode AFM on a MultiMode 8 system (Bruker FRANCE S.A.S., Strasbourg, Germany), as well as SEM using a Thermo Scientific Scios2 (Brno, Czech Republic) instrument. Point defects were introduced in the atomic structure of the graphene overlayer (sample #2) by 5 s of O_2_ plasma treatment (Zepto cleaner, Diener electronic, Ebhausen, Germany) at 50 W, followed by annealing (400 °C) in Ar gas for 60 min.

### 2.2. UV–Vis Spectroscopy and Vapor Sensing Setup

Optical reflectance and vapor sensing measurements were carried out by mounting the samples in an airtight aluminum box covered with a fused silica window to provide UV transmission. The samples were illuminated using an Avantes AvaLight DH-S-BAL light source. The initial reflectance of the samples in synthetic air was measured with an Avantes HS 1024 × 122TEC spectrometer (Avantes BV, Apeldoorn, The Netherlands) by capturing the specularly reflected signal (measured under 15°). For vapor sensing experiments, five types of volatile vapors were passed through the cell’s gas inlet and vented through the outlet: acetone, ethanol, 2-propanol, toluene, and water (analytical-grade, VWR International Ltd., Radnor, PA, USA). Vapor concentration was adjusted by switching digital mass flow controllers (Aalborg DFC, Aalborg Instruments & Controls, Inc., Orangeburg, NY, USA) to pass synthetic air (Messer, 80% N_2_, 20% O_2_) and saturated volatile vapors through gas bubblers in the required ratio. During the measurements, a constant gas flow of 1000 mL/min was sustained through the cell. Vapor detection experiments were performed by varying the concentration and type of test vapor while tracking spectral variations over time. To purge the cell, a 20 s vapor flow at a given concentration was followed by a 60 s flow of synthetic air. In addition, before introducing the next vapor type, a 120 s purge was used to recover the initial reflectance of the sample.

## 3. Results and Discussion

**Figure 1:**
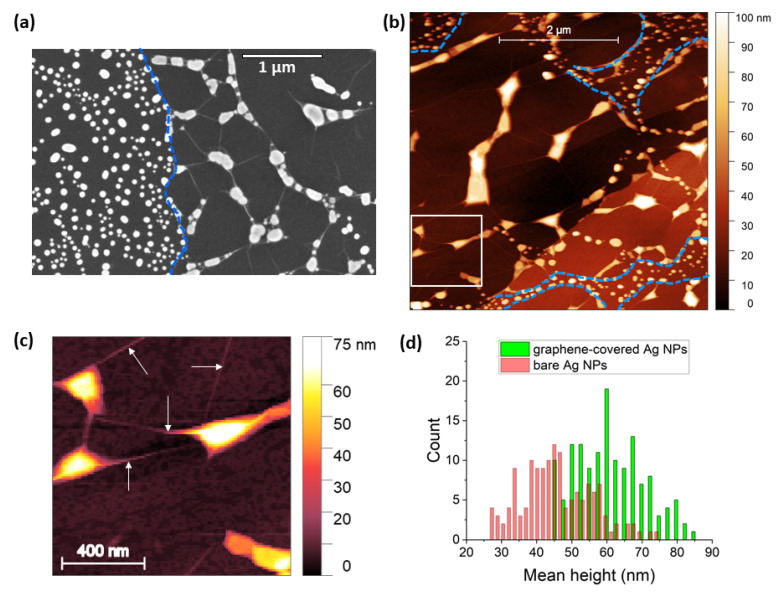
(**a**) SEM image of bare Ag NPs (left) and Ag NPs covered with graphene (right). The edge of graphene is marked with a blue dashed line as a guide for the eye. (**b**) AFM image of graphene-encapsulated Ag NPs. Discontinuities in the graphene overlayer and areas with bare Ag NPs are demarcated with blue dashed lines. Several graphene-covered nanoparticles are demarcated with a white square and shown in the enlarged image in (**c**). Here, wrinkling of the graphene overlayer is observed (arrows). (**d**) The mean height distribution of graphene-covered (green) and bare Ag NPs (red) was measured on 142 NPs in both cases.

Tapping-mode AFM and SEM were used to characterize the formed nanoparticles. The graphene-covered Ag nanostructures had elongated shapes (Figure 1a,b), with an average height of 61 nm. Typical nanoparticles that are completely sandwiched between graphene and HOPG are shown in Figure 1c. Graphene wrinkles also developed near these NPs. In contrast, smaller NPs formed in areas with graphene discontinuities. These bare NPs had a rather disk-like shape, with an average height of 45 nm. The mean height distribution of both graphene-covered and bare Ag NPs is shown in Figure 1d. 

**Figure 2:**
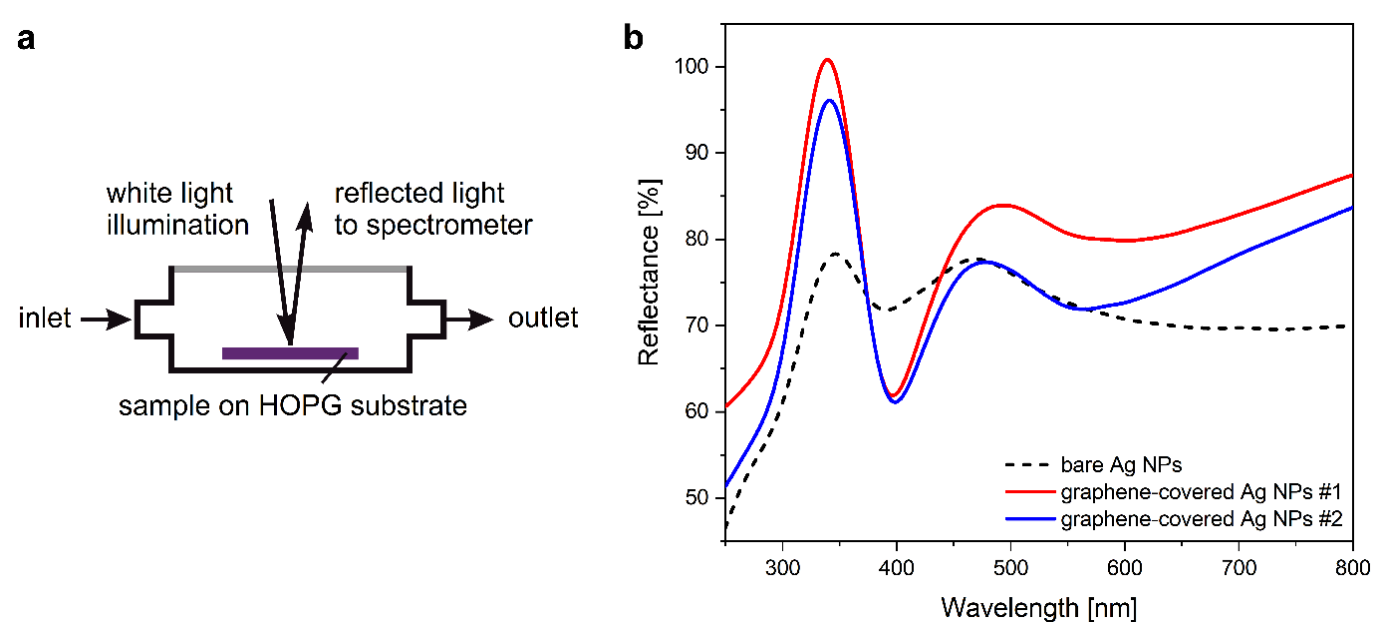
(**a**) Schematic drawing of the aluminum cell used in vapor sensing experiments. (**b**) Optical reflectance spectra of bare (dashed) and graphene-covered Ag NPs measured in air (samples #1 and #2 are similar).

The optical properties of such nanoparticle ensembles were studied by UV–Vis reflectance spectroscopy. For this, the samples were fixed inside an aluminum cell with a quartz window, as illustrated in Figure 2a, and a synthetic air atmosphere. Specular reflection was applied at 15° degrees between the two optical fibers used for illumination and light collection. Typical reflectance spectra are shown in Figure 2b, where the LSPR is observed as prominent minima near 400 nm. As a reference, we used a bare HOPG surface. In vapor sensing experiments, the spectral response was the shift in this sharp LSPR, and it was characterized by dividing the measured spectrum with the reference spectrum of Figure 2b, measured in synthetic air. 

Note that the bare Ag NPs had a low LSPR intensity with a reflectance minimum at 390 nm, while the LSPR wavelength of graphene-covered Ag NPs was at 397 and 399 nm, for samples #1 and #2, respectively. The redshift in the spectra of graphene-covered nanoparticles, compared to the spectrum of bare Ag NPs, is related, on one hand, to the *n*-type doping of graphene by electrostatic contact with silver [41], and, on the other hand, the graphene-covered NPs are larger than the bare NPs (see Figure 1), resulting in a redshift [42]. Moreover, the observed redshift can be partly ascribed to the increased effective refractive index of the medium surrounding the NPs [34]. As the LSPR wavelength is sensitive to the composition of the nearby atmosphere, it can be efficiently used for the detection of VOC molecules adsorbed on the surface. 

**Figure 3:**
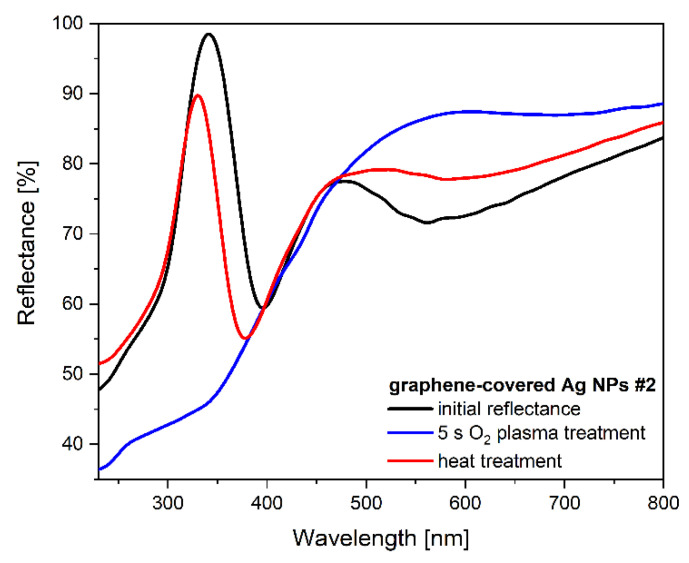
Optical reflectance spectra of the graphene-covered Ag NP sample #2 before (black) and after (blue) O_2_ plasma treatment. The LSPR is recovered (red) by annealing at 400 °C.

The adsorption of organic molecules to graphene is influenced by the presence of defects, as shown by recent calculations [43]. This, in turn, can affect the sensing properties of samples having a defected graphene overlayer. To investigate this, we introduced defects in sample #2 using O_2_ plasma for 5 s. Such plasma treatment induced individual, point-like defects (vacancies) separated by several nanometers, as shown in Figure S1. Although the exposure to plasma was short, the nanoparticles were also affected. The reflectance measured after plasma treatment did not show a well-defined LSPR; it rather showed a significant decrease in the reflectance in the near-UV (Figure 3, spectrum in blue). 

This abrupt change in the optical characteristics is ascribed to the silver reacting with oxygen, and the formation of AgO or Ag_2_O phases [44]. These oxide phases were successfully decomposed by applying subsequent annealing of sample #2 at 400 °C in an inert atmosphere, which also resulted in the recovery of the metallic Ag NPs. This is expressed by the recovered resonance, as shown in Figure 3 (spectrum in red). Note that after annealing, the new LSPR was measured at a lower wavelength (385 nm). The applied annealing also stabilized the initially produced vacancy-type defects by dangling bond saturation either through the adsorption of molecules, or via the formation of non-hexagonal carbon rings (pentagons, heptagons, etc.) [45].

**Figure 4:**
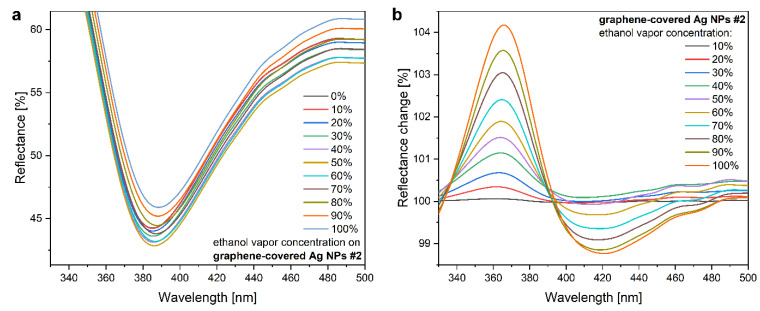
(**a**) LSPR shift of graphene-covered Ag NPs (sample #2) for different concentrations of ethanol vapor. The initial spectrum (0%) measured in synthetic air was used as a reference. (**b**) Reflectance change spectra, calculated from the same measurements (see main text).

**Figure 5:**
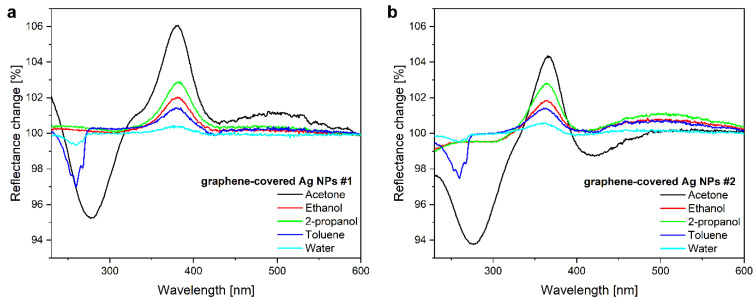
Reflectance change spectra of graphene-covered Ag NP samples when saturated vapors of acetone, ethanol, 2-propanol, toluene, or water are applied. The optical responses are substance-specific for both (**a**) as-prepared (#1) and (**b**) O_2_ plasma-treated (#2) samples.

**Figure 6:**
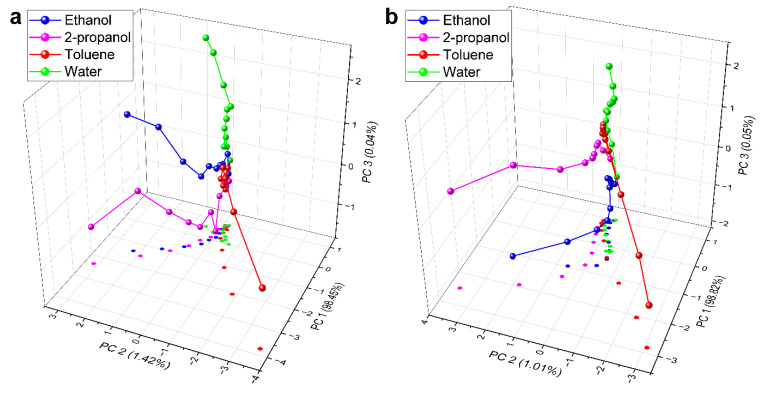
PCA scores plots of ethanol, 2-propanol, toluene, and water, calculated for the two graphene-covered Ag NP samples. Slightly different vapor sensing properties can be seen in (**a**) sample #1 (pristine graphene overlayer), compared to (**b**) sample #2 (defected graphene overlayer). The cumulative variance of the first three PCs was above 99% for both samples.

In the following, we used both graphene-covered Ag NP samples in VOC-sensing experiments: sample #1 with pristine graphene, and sample #2 with a defected graphene overlayer. Saturated vapors of acetone, ethanol, 2-propanol, toluene, and water were diluted with synthetic air in ten different concentrations and the resulting wavelength shift of the LSPR peak was measured. As a control experiment, a bare HOPG surface was used in the same vapor sensing measurement, which resulted in only a slight decrease in the intensity as the vapor concentration was increased (see Figure S2). The effect is attributed to the adsorption of vapors onto the HOPG surface [46]. This minor baseline shift was measured in all graphene-covered Ag NP samples too, which is corrected in Figure 4 and Figure 5, but the original data sets were used in the more detailed analysis shown later in Figure 6. The vapor concentrations were applied in increasing order, and the observed LSPR shift was proportional to the concentration for all VOCs. As an example, the data obtained for ethanol are shown in Figure 4a. Since the LSPR shifts were rather small changes compared to the LSPR intensity, it was more convenient to use the reflectance change spectra (Figure 4b) defined as Δ*R* = (*R* / *R*_0_) × 100%, where *R*_0_ is the initial reflectance of the sample in synthetic air. The calculated reflectance change spectra clearly showed an increasing signal as a function of the vapor concentration. A higher number of adsorbed molecules increased the effective refractive index of the medium more significantly. A positive peak followed by a negative peak represent a redshift of the LSPR. In this way, the original small changes in the LSPR could be effectively compared for all investigated samples and substances.

The optical responses of graphene-covered Ag NPs obtained for saturated vapors are plotted in Figure 5. Although both samples #1 and #2 showed similar responses, the reflectance change spectra were found to be unique for each VOC, when compared to each other. For example, in addition to the induced LSPR shift, the UV absorption of acetone and toluene vapors was also observed.

To investigate in more detail the VOC sensing data, principal component analysis (PCA) of the measured data sets was carried out and the resulting score plots were compared. As both samples showed a much higher response to acetone compared to the other four substances, and because the UV absorbance of this vapor was in the range of the LSPR, the data on acetone were omitted from the analysis. The PCA score plots of ethanol, 2-propanol, toluene, and water are shown in Figure 6. The cumulated variance of the first three PCs was above 99% for both samples.

**Figure 7:**
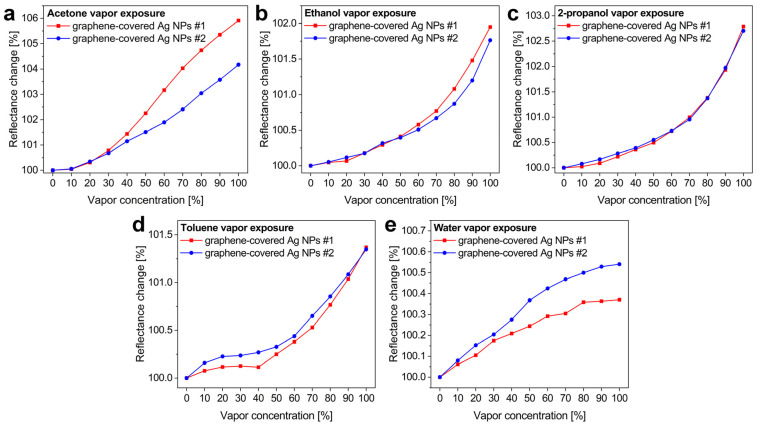
Maximal peak intensities in the reflectance change spectra as a function of vapor concentration. For each applied vapor, the optical responses of samples #1 and #2 are compared. (**a**) Acetone, (**b**) ethanol, (**c**) 2-propanol, (**d**) toluene, and (**e**) water vapors were applied.

Here, each vapor had a unique trajectory. To facilitate the comparison, the data points were projected on the PC1–PC2 planes of the graphs. All trajectories originated from a common point, which was the data point of the initial synthetic air spectrum. Away from this, as the concentration of the vapors increased, the calculated points branched apart, resulting in a clear separation of the trajectories. This showed that the VOC detection by the graphene-covered Ag NPs was substance-specific. One can see on the PC1–PC2 projected plane that the third dimension is needed to properly separate the signal of ethanol and 2-propanol, as the trajectories are overlapping in the 2D projection. Furthermore, to observe the effect of defects in the graphene overlayer, we compared the maximal intensities of the peak (near the LSPR) in the reflectance change spectra to the maximal intensities obtained with the pristine graphene overlayer. These maximal peak intensities are plotted as a function of concentration in Figure 7, for each applied vapor. 

One can observe that there were differences in the maximal responses of the two samples, especially for acetone, water, and toluene. The defected graphene overlayer increased the sensitivity to water and toluene. This agrees with recent calculations showing that toluene [43] and water [47,48] adsorb better to graphene with defects. In comparison, exposure to ethanol and 2-propanol resulted in similar optical responses, while acetone even gave a reduced signal on defected graphene. To explain such changes in the sensitivity, one must consider the interaction between the VOC molecules, defected graphene, and the underlying Ag NPs as well, since possible charge redistribution effects can result in a slight, opposite shift in the LSPR, reducing the main effect of the refractive index change. This calls for theoretical calculations, which were beyond the scope of the current work. Nonetheless, as demonstrated in Figure 7, the sensitivity to certain vapors can be tuned with structural defects, which can be further explored in the development of selective VOC detectors.

## 4. Conclusions

We investigated the morphology, optical, and vapor sensing properties of graphene-covered Ag NPs elaborated directly on HOPG substrates. We showed that the prepared hybrid nanostructures displayed pronounced optical responses upon exposure to organic vapors (acetone, ethanol, 2-propanol, toluene, and water). The observed concentration-dependent shifts in the LSPR were substance-specific, as demonstrated by reflectance change spectra and PCA. We further showed that, by introducing structural defects in the graphene overlayer, the optical response of the graphene/Ag NPs increased for toluene and water, while it decreased for acetone detection. These findings can stimulate further research towards increasing the selectivity in VOC sensing.

## Data Availability

The data presented in this study are available on request from the corresponding author.

## Supplementary Materials

The following supporting information can be downloaded at: https://www.mdpi.com/article/10.3390/nano12142473/s1, Figure S1: STM image of a HOPG substrate exposed to 5 seconds of O_2_ plasma, Figure S2: Reflectance of bare HOPG surface when different concentrations of ethanol vapor were applied.
