# Supplementary material for: Graphene-Encapsulated Silver Nanoparticles for Plasmonic Vapor Sensing
Source: Nanomaterials (Basel). 2022 Jul 19;12(14):2473. doi: 10.3390/nano12142473 (PMC9319566; doi:10.3390/nano12142473)
Supplement: Supplementary file 1 [file nanomaterials-12-02473-s001.zip › nanomaterials-1800117-supplementary.pdf]

nanomaterials

MDPI

Supplementary Information

Graphene-Encapsulated Silver Nanoparticles for Plasmonic Vapor Sensing

Gábor Piszter, György Molnár, András Pálinkás and Zoltán Osváth *

Centre for Energy Research, Institute of Technical Physics and Materials Science, 1121 Budapest, Hungary; gabor.piszter@ek-cer.hu (G.P.); gyorgy.molnar@ek-cer.hu (G.M.); andras.palinkas@ek-cer.hu (A.P.)
* Correspondence: zoltan.osvath@ek-cer.hu

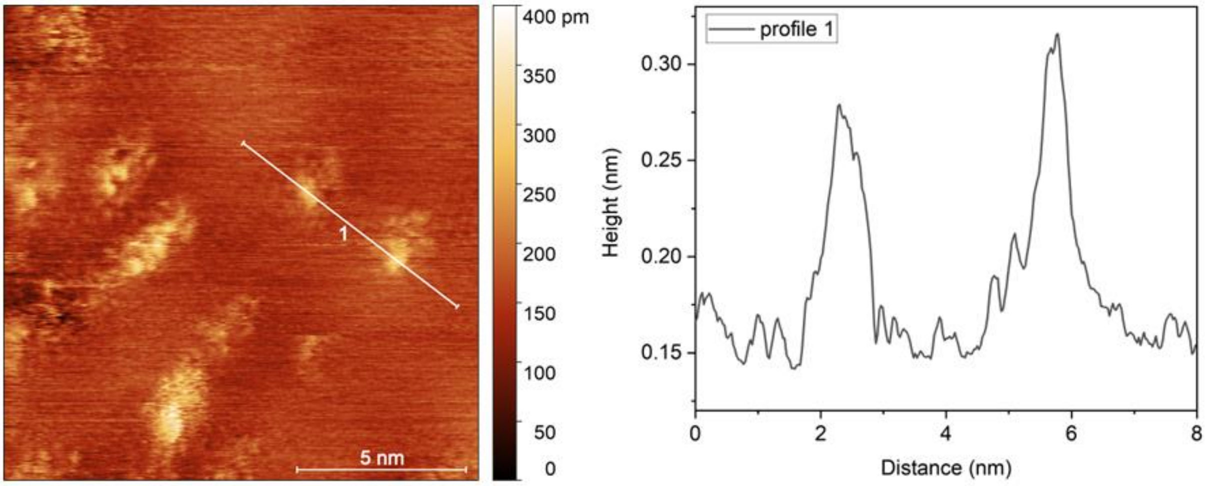

Figure S1. Left: STM image of a HOPG substrate exposed to 5 seconds of O_2 plasma. Individual defects appear as hillock-like protrusions due to local changes in the density of states. Right: Height profile of line section "1" shown in the STM image.

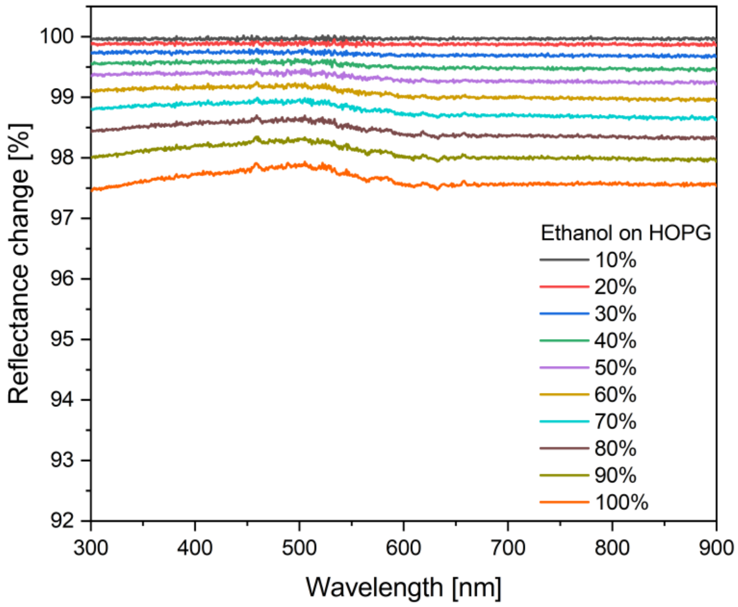

Figure S2. Reflectance of bare HOPG surface when different concentrations of ethanol vapor were applied. One can note a slight decrease in the intensity as the vapor concentration is increased. The effect is attributed to the adsorption of vapors onto the HOPG.
